# Impact of cardiac surgery associated acute kidney injury on 1-year major adverse kidney events

**DOI:** 10.3389/fneph.2023.1059668

**Published:** 2023-04-24

**Authors:** Alícia Molina Andújar, Victor Joaquin Escudero, Gaston J. Piñeiro, Alvaro Lucas, Irene Rovira, Purificación Matute, Cristina Ibañez, Miquel Blasco, Luis F. Quintana, Elena Sandoval, Marina Chorda Sánchez, Eduard Quintana, Esteban Poch

**Affiliations:** ^1^ Nephrology and Kidney Transplantation Department, Hospital Clínic, Barcelona, Spain; ^2^ Faculty of Medicine, University of Barcelona, Barcelona, Spain; ^3^ Institut d’investigacions biomèdiques Agustí Pi i Sunyer (IDIBAPS), Barcelona, Spain; ^4^ Anesthesiology Department, Hospital Clinic, Barcelona, Spain; ^5^ Cardiovascular Surgery Department, Hospital Clinic, Barcelona, Spain; ^6^ Perfusion Department, Hospital Clinic, Barcelona, Spain

**Keywords:** score, chronic kidney disease (CKD), major adverse kidney events (MAKE), acute kidney injury (AKI), cardiac surgery

## Abstract

**Background:**

The incidence of acute kidney injury following cardiac surgery (CSA-AKI) is up to 30%, and the risk of chronic kidney disease (CKD) has been found to be higher in these patients compared to the AKI-free population. The aim of our study was to assess the risk of major adverse kidney events (MAKE) [25% or greater decline in estimated glomerular filtration rate (eGFR), new hemodialysis, and death] after cardiac surgery in a Spanish cohort and to evaluate the utility of the score developed by Legouis D et al. (CSA-CKD score) in predicting the occurrence of MAKE.

**Methods:**

This was a single-center retrospective study of patients who required cardiac surgery with cardiopulmonary bypass (CPB) during 2015, with a 1-year follow-up after the intervention. The inclusion criteria were patients over 18 years old who had undergone cardiac surgery [i.e., valve substitution (VS), coronary artery bypass graft (CABG), or a combination of both procedures].

**Results:**

The number of patients with CKD (eGFR < 60 mL/min) increased from 74 (18.3%) to 97 (24%) within 1 year after surgery. The median eGFR declined from 85 to 82 mL/min in the non-CSA-AKI patient group and from 73 to 65 mL/min in those with CSA-AKI (*p* = 0.024). Fifty-eight patients (1.4%) presented with MAKE at the 1-year follow-up. Multivariate logistic regression analysis showed that the only variable associated with MAKE was CSA-AKI [odds ratio (OR) 2.386 (1.31–4.35), *p* = 0.004]. The median CSA-CKD score was higher in the MAKE cohort [3 (2–4) vs. 2 (1–3), p < 0.001], but discrimination was poor, with a receiver operating characteristic curve (AUC) value of 0.682 (0.611–0.754).

**Conclusion:**

Any-stage CSA-AKI is associated with a risk of MAKE after 1 year. Further research into new measures that identify at-risk patients is needed so that appropriate patient follow-up can be carried out.

## 1 Introduction

Acute kidney injury (AKI) is a sudden loss of kidney function that, from start to finish, occurs in less than 7 days. It is well known from experimental models that, depending on the severity of AKI, some tubule cells are irreversibly lost and replaced by renal progenitor cells. Tubules regenerating after AKI may fail to differentiate and exhibit profibrotic paracrine activity before they become atrophic, so these mechanisms of loss and maladaptive repair imply post-AKI chronic kidney disease (CKD) and a reduction of kidney lifespan (1, 2). In addition, clinical data suggest that AKI at any stage is an independent risk factor for CKD and end-stage CKD (ESCKD) (3). Although the connection between AKI and CKD is well established, it was not until 2017 that the Acute Disease Quality Initiative (ADQI) reached a consensus and defined acute kidney disease (AKD) as disease developing in the period between 7 and 90 days after AKI, which led to the design of studies focusing on interventions in this period, with the aim of preventing CKD after AKI (1).

The incidence of AKI following cardiac surgery (CSA-AKI) is up to 30%, and 2%–5% of patients require renal replacement therapy (RRT) during an AKI episode. CSA-AKI increases the risk of death during admission, which can increase to 50% when there is a need for RRT (4). Given the high incidence of AKI in this controlled scenario, studies have focused on the incidence of *de novo* CKD [defined as eGFR < 60 mL/min] after cardiac surgery. In 2017, Legouis D et al. studied a cohort of 4,791 patients and found that the risk of CKD was higher in patients who had experienced CSA-AKI than in the AKI-free population (5).

Despite the link between AKI and CKD, information about AKI (even for those patients with a need for RRT) is not always provided in the discharge documentation, which makes it difficult for primary care doctors to improve their kidney function follow-up. This issue was recently reviewed by Ostermann et al. (6). Among the AKI patients who received RRT in intensive care units (ICUs) in the UK, the development of AKI and the need for RRT were mentioned in 85% and 82% of critical care discharge letters, respectively, and the monitoring of kidney function post discharge was recommended in only 36.3% of hospital discharge summaries (6).

Providing clinicians with tools to identify patients at risk of CKD after AKI should be a key priority. With this in mind, Legouis D et al. developed a prediction score for *de novo* CKD (defined as e GFR< 60 mL/min) 1 year after cardiac surgery that was found to have fair accuracy in a validation cohort [receiver operating characteristic curve (AUC) value of 0.78]. The score comprises preoperative eGFR by Modification of Diet in Renal Disease formula (MDRD) < 80 mL/min (1 point), age > 65 years (1 point), transplant or aortic surgery (2 points), aortic clamping time > 50 minutes (1 point), and AKI stage one (1 point) and AKI stage 2 or 3 (2 points) (7).

With the aim of including all clinically meaningful renal endpoints in AKI clinical trials, the concept of major adverse kidney events (MAKE) was introduced. This composite endpoint comprises persistently impaired renal function (i.e., a 25% or greater decline in eGFR), new hemodialysis, and death. It has been proposed as a way to improve the capacity to understand AKI and provide a means of comparing different interventions (8).

The aim of our study was to assess the incidence of MAKE 1 year after cardiac surgery and its risk factors and, as a secondary objective, to evaluate the utility of the score developed by Legouis D et al. (CSA-CKD score) in the prediction of MAKE 1 year after surgery, and in so doing to shed light on potential tools for the identification of at-risk patients that require particular follow-up.

## 2 Materials and methods

We conducted a unicentric retrospective study of patients admitted to Hospital Clínic de Barcelona for cardiac surgery with cardiopulmonary bypass (CPB) from January 2015 to December 2015, with a 1-year follow-up after the intervention. The inclusion criteria were patients over 18 years old who had undergone cardiac surgery [i.e., valve substitution (VS), coronary artery bypass graft (CABG), or a combination of both procedures] and who were in need of a CPB. Patients with chronic kidney diseases at any stage were included. However, patients who were already undergoing chronic dialysis therapy, renal transplant recipients, and those who had had an AKI immediately prior to surgery were not included in the study. In addition, patients who had undergone emergent surgeries, intra-aortic balloon pump (IABP) users, patients who died during surgery or admission, and patients with endocarditis were excluded. The Ethics Committee of our institution approved the study (Reg. HCB/2019/0959)

### 2.1 Data collection

Clinical, epidemiological, and laboratory variables were collected from our institution’s Electronic Health Records (EHR), SAP^®^. For each patient, data on medical history, surgery characteristics, intraoperative variables, 24-hour monitoring period in the intensive care unit (ICU), and renal function evolution until discharge and at the 1-year follow-up were collected. Data pertaining to the duration and type of RRT for those patients who required it were also recorded.

Baseline variables included sex, age, medical history, anthropometric variables, Charlson Index Comorbidity Score, creatinine and hemoglobin values before surgery, smoking status, and ejection fraction. Surgical variables included the type of surgery, need for transfusion, ischemia time, extracorporeal circulation time, furosemide or ultrafiltration requirements, and the use of vasopressors, vasodilators, or inotropic drugs. Variables recorded during the first 24 hours included renal function, need for transfusion, use of vasopressors, vasodilators, or inotropic drugs, and need for iodinated contrast media. Information on MAKE was collected 1 year after surgery.

Leicester score (LS), Cleveland Clinic score (CCS), and Euroscore II were calculated for each patient using the information collected during pre-anesthetic visits and/or patient admission reports. CSA-CKD scores were calculated using information from the reports on patient admission and discharge.

Data on new AKI episodes occurring in the first year after discharge from cardiac surgery were extracted from the EHR.

### 2.2 Definitions

CSA-AKI was defined in accordance with the Kidney Disease Improving Global Outcomes (KDIGO) criteria, i.e., as an increase in serum creatinine (sCr) of ≥ 0.3 mg/dL within 48 hours or of ≥ 1.5- to 2-fold from baseline within 1 week after surgery. Owing to the nature of this study, urinary output criteria were not included. Moderate AKI was defined as a 2.0- to 2.9-fold increase in sCr from baseline, and severe AKI was defined as a 3-fold increase in sCr from baseline or an increase of 0.5 mg/dL if the sCr level was ≥ 4.0 mg/dL at baseline or at the beginning of RRT. The baseline sCr level for CSA-AKI measurements was taken as the value obtained 24 hours before surgery. The duration of AKI was regarded as being from the AKI diagnosis until the sCr level returned to baseline (± 0.3 mg/dL).

MAKE within 1 year of cardiac surgery discharge was defined as persistent renal function decline (i.e., a > 25% decline in eGFR), a new requirement for hemodialysis, or death. Baseline and 1-year eGFR values were calculated using the Chronic Kidney Disease Epidemiology Collaboration (CKD-EPI) formula. The baseline eGFR for the 1-year MAKE assessment was taken as the value obtained in the pre-anesthetic chart or, if this was not available, as the value obtained 24 hours before surgery.

### 2.3 Statistics

The study variables are expressed as mean ± standard deviation (SD) if normally distributed, and as medians and interquartile ranges (IQRs) if not. Categorical variables are expressed in terms of absolute values (n) and relative frequency (%). p-values less than 0.05 were considered significant. Variables associated with a risk of MAKE after 1 year were assessed by logistic regression in univariate analysis, and those with statistical significance or clinical relevance were included in the multivariate analysis. We determined the overall performance of the CSA-CKD score by calculating the AUC and carrying out the Hosmer–Lemeshow goodness-of-fit test to assess its discrimination and calibration, respectively. A p-value above 0.05 indicated acceptable calibration. The statistical analysis was conducted using SPSS software, v.25 (SPSS Inc, Chicago, IL, USA).

## 3 Results

### 3.1 Characteristics of the population

A total of 404 patients met the inclusion criteria and completed the 1-year follow-up period. Baseline characteristics are depicted in **Table 1**. The majority of patients (63.4%) were men, and the median age at the time of surgery was 69 years (IQR 61–76 years). Hypertension was the most prevalent comorbidity, followed by diabetes and obesity (presenting in 76.5%, 35.4%, and 30.7% of patients, respectively). Peripheral vascular disease was diagnosed in only 8.9% of patients. The median baseline sCr was 0.9 mg/dL (IQR 0.73–1.05 mg/dL), and 18.3% of the patients had an eGFR of < 60 mL/min. Anemia (hemoglobin level < 120 g/L) was present in 18.6% of patients before cardiac surgery. The most common procedure was VS (46%), followed by CABG (37.4%). Intraoperative variables and AKI scores/surgical risk are included in **Supplementary Material Table 1**. It should be noted that 78 out of the 404 patients (19.3%) had a cardiopulmonary bypass time of over 120 minutes.

**Table 1:** Baseline characteristics.

N = 404	N (%)/median (IQR)/mean ± SD
Sex (% men)	256 (63.4)
Age (years) ≥ 75	69 (61–76) 122 (30.2)
History of smoking	188 (46.5)
Diabetes Diabetes with insulin therapy	143 (35.4) 38 (9.4)
Hypertension	309 (76.5)
Charlson Comorbidity Index score	4 (3–5)
BMI (kg/m^2)^ BMI ≥ 30	28.25 **±** 4.47 124 (30.7)
Anemia Hemoglobin (g/L) Hematocrit (%)	75 (18.6) 134 (123–144) 39 (36–42)
Peripheral vascular disease	36 (8.9)
Low ejection fraction (< 40%)	40 (9.9)
Creatinine (mg/dL) eGFR(mL/min) eGFR< 60 mL/min CKD SIII CKD SIV	0.9 (0.73–1.05) 81 (66–92) 74 (18.3) 63 (15.6) 10 (2.5)
Previous cardiac surgery	43 (10.6)
Procedure	Valve surgery: 186 (46) CABG: 151 (37.4) Valve + CABG: 67 (16.6)

BMI, body mass index; eGFR, estimated glomerular filtration rate; CKD, chronic kidney disease; IQR, interquartile range; SD, standard deviation; CABG, coronary artery bypass grafting.

One hundred and forty-seven (36.4%) patients had CSA-AKI, which for the majority of patients was stage 1 (63.3%) and started within the first 24 hours after surgery. The median duration of AKI (i.e., the time from AKI diagnosis until sCr levels returned to baseline value ± 0.3 mg/dL) was 3 days (IQR 1–6 days), and 10 patients (2.5%) required RRT. Additional information pertaining to patients’ CSA-AKI characteristics is provided in **Supplementary Material Table 2**. The median sCr level at discharge was 0.86 mg/dL (IQR 0.69–1.04 mg/dL), and the median eGFR was 84 mL/min (IQR 64–95 mL/min). Twenty-nine out of 147 patients with AKI (19.7%) had persistent renal dysfunction decline at discharge (i.e., a > 25% decline in eGFR) but none of these patients was receiving RRT.

### 3.2 Renal function and MAKE 1 year after cardiac surgery

**Figure 1:**
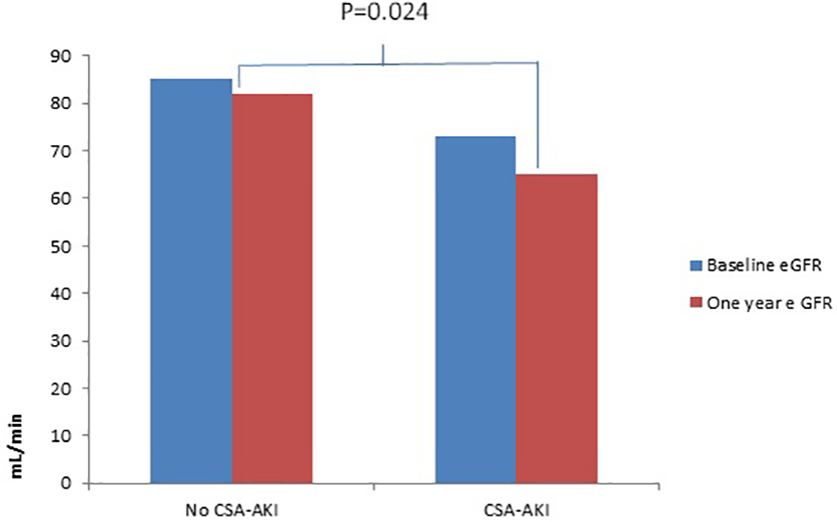
Differences in eGFR at 1-year follow-up between patients who had CSA-AKI and those who did not. CSA-AKI, cardio surgery associated acute kidney injury; e GFR, estimated glomerular filtration rate.

In the overall cohort, sCr levels and eGFR at 1 year were similar to those at baseline [0.93 mg/dL (IQR 0.78–1.10 mg/dL), 78 mL/min (IQR 61–90 mg/dL)], but when the cohort was divided between those who had AKI and those who did not, the eGFR declined from 85 to 82 mL/min and from 73 to 65 mL/min (*p* = 0.024) in the non-AKI and AKI groups, respectively (**Table 2** and **Figure 1**). The number of patients with CKD (eGFR < 60 mL/min) increased from 74 (18.3%) to 97 (24%) within 1 year after surgery.

**Table 2:** Changes in sCr level and eGFR in the overall cohort, CSA-AKI, and no-CSA-AKI cohort.

	Overall cohort N = 404 median (IQR)	CSA-AKI *n *= 147 median (IQR)	No CSA- AKI *n* = 257 median (IQR)
Baseline sCr level (mg/dL)	0.9 (0.73–1.05)	0,97 (0.79–1.22)	0.86 (007–1.01)
1-year sCr level (mg/dL)	0,93 (0.78–1.10)	1.02 (0.86–1.28)	0.89 (0.76–1.08)
Baseline eGFR (mL/min)	81 (66–92)	73 (54–87)	85 (71–95)
1-year eGFR (mL/min)	78 (61–90)	65 (51–83)	82 (68–93)

IQR, interquartile range; CSA-AKI, cardiac surgery associated acute kidney injury; eGFR, estimated glomerular filtration rate.

**Figure 2:**
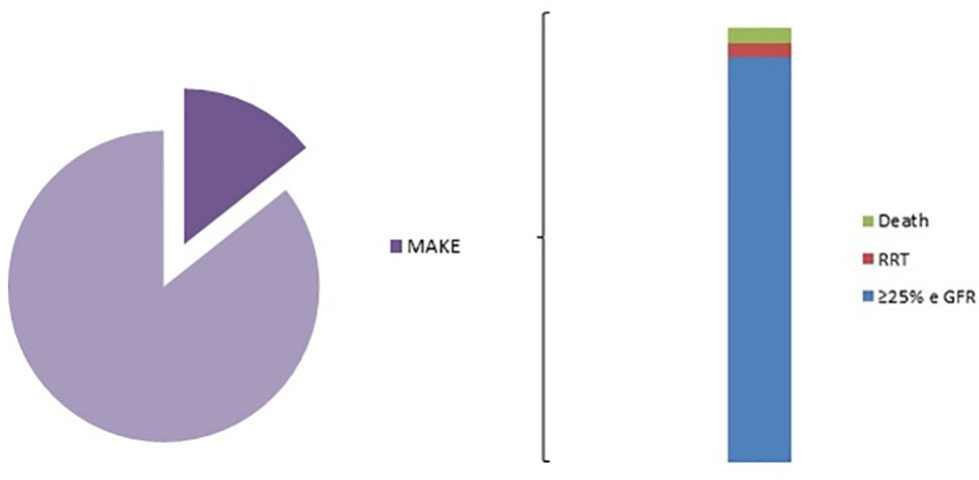
MAKE distribution. MAKE, major adverse kidney events. RRT, Renal Replacement Therapy; e GFR, estimated glomerular filtration rate.

Fifty-eight (14.36%) patients had experienced MAKE within 1 year after surgery. Incidences of MAKE included a decline by ≤ 25% in eGFR in 54 patients, the need for RRT in two patients, and the death of two patients (**Figure 2**). The association of CSA-AKI with MAKE was assessed in a univariate logistic regression analysis, including any-stage CSA-AKI, long CSA-AKI, and CSA-AKI with the need for RRT, and the three forms of CSA-AKI were statistically associated with the outcome (**Table 3**).

**Table 3:** Univariate analysis of CSA-AKI as a risk factor for 1-year MAKE.

	Total (N = 404)	MAKE (*n* = 58)	No MAKE (*n* = 346)	OR (95% CI)	*p*-value
Any-stage CSA-AKI (%)	147 (36.4)	34 (58,6)	113 (32.7)	2.921 (1.654 to 5.159)	< 0.001
Long CSA-AKI (>3 days)(%)	58 (14.4)	20 (34.5)	38 (11)	4.266 (2.254 to 8.072)	< 0.001
RRT CSA-AKI (%)	10 (2.5)	4 (6.9)	6 (1.7)	4.198 (1.147 to 15.36)	0.003

CSA-AKI, cardiac surgery associated acute kidney injury; OR, odds ratio; RRT, renal replacement therapy.

A univariate analysis of baseline characteristics was performed to identify baseline risk factors that could be also associated with the risk of MAKE within 1 year after surgery so that these could be included in the multivariate analysis. Among the included baseline variables, patients who were associated with MAKE 1 year after surgery were over 75 years of age [odds ratio (OR) 2.12 (1.2–3.74), *p* = 0.01], or having arterial hypertension [OR 2.42 (1.07–5.59), *p* = 0.034], or preoperative anemia [OR 2.27 (1.22–4.25), *p* = 0.01]. As for renal function, an eGFR of < 60 mL/min was considered almost statistically significant [OR 1.85 (0.98–3.52), *p* = 0.059] (**Table 4**). Relatedly, median Charlson Comorbidity Index Scores was higher for patients who had experienced MAKE [4.5 (3–6)] than in those who had not [4 (3–5)].

**Table 4:** Univariate analysis of baseline risk factors for 1-year MAKE.

N (%)/median (IQR)/mean+/-SD	MAKE (*n* = 58)	No MAKE (*n* = 346)	OR (95% CI)	*p*-value
**Male sex**	34 (58.6)	222 (64.4)	0.791 (0.449–1.395)	*p* = 0.418
**Age ≥ 75 years**	26 (44.8)	96 (27,7)	2.116 (1.198–3.736)	*p* = 0.010
**Smoking status**	28 (48.3)	160(46.2)	1.080 (0.616–1.894)	*p* = 0.788
**Diabetes**	23 (39.7)	120 (34.7)	1.238 (0.699–2.190)	*p* = 0.464
**Hypertension**	51 (87.9)	259 (74.9)	2.4247(1.071–5.593)	*p* = 0.034
**BMI ≥ 30**	17 (29,3)	107 (30.9)	0.94 (0.503–1.755)	*p* = 0.845
**Anemia**	7 (12.1)	57 (16.5)	2.27 (1.217–4.246)	*p* = 0.01
**Peripheral vascular disease**	6 (10.5)	30 (8,7)	1.215 (0.482–3.063)	*p* = 0.679
**EF < 40%**	8 (13.8)	32 (9.2)	1,57 (0.684–3.601)	*p* = 0.287
**EGFR< 60 mL/min** ** CKD SIII** ** CKD SIV**	16 (27.6) 13 (22.4) 3 (5.2)	59 (17,1) 52 (15) 7 (2)	FG<60: 1.853 (0.977–3.516)	*p* = 0.059
**Past cardiac surgery**	7 (12.1)	36 (10.2)	1.178 (0.497–2.790)	*p* = 0.709
**Procedure:** **VS** **CABG** **VS +CABG**	35 (60.3) 16 (27.6) 7 (12.1)	151 (43.6) 135 (39) 60 (17.3)	CABG: 0.595 (0.322–1.101)	*p* = 0.098

IQR, Interquartile range; MAKE, major adverse kidney events; OR, odds ratio; BMI, body mass index; EF, ejection fraction; CKD, chronic kidney disease; VS, valve substitution; CABG, coronary artery bypass graft.

A multivariate logistic regression analysis was performed with 1-year MAKE within 1 year after surgery as a dependent variable and any-stage CSA-AKI (with the statistically significant baseline variables being the patient having arterial hypertension, preoperative anemia, or being aged > 75 years) and CKD (with the statistically significant baseline variable being an eGFR of < 60 mL/min) as clinically relevant independent variables. In that analysis, the only variable that was still associated with MAKE 1 year after surgery was any-stage CSA-AKI [OR 2.386 (1.31–4.35), *p* = 0.004) **Table 5**.

**Table 5:** Multivariate analysis of risk factors associated to 1-year MAKE.

	OR	95% CI	*p*-value
Age > 75 years	1,657	0.914–3.006	0.096
AHT	1.895	0.811–4.430	0.140
Anemia	1.799	0.932–3.473	0.080
Any-stage CSA-AKI	2.386	1.31–4.346	0.004
Baseline eGFR < 60 mL/min	1.112	0.557–2.223	0.763

AHT, arterial hypertension; CSA-AKI, cardiac surgery associated acute kidney injury; MAKE, major adverse kidney events; eGFR, estimated glomerular filtration rate.

### 3.3 CSA-CKD score

**Figure 3:**
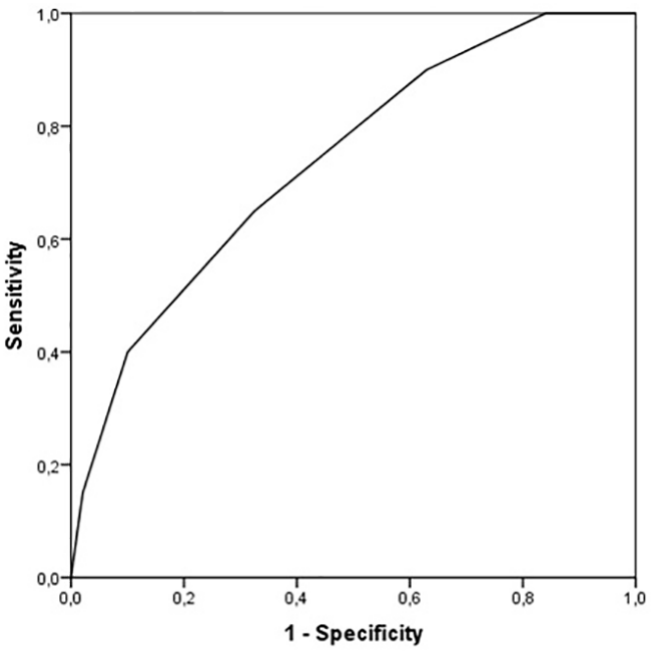
Receiver operating characteristic curve of CSA-CKD score for CKD. CSA-CKD, Cardiac surgery associated chronic kidney disease; CKD, chronic kidney disease.

Because the CSA-CKD score study validation was performed in patients without pre-existing CKD (i.e., those with an eGFR of < 60 mL/min) to predict the likelihood of CKD after 1 year, we first assessed the performance of the score in the selected population with an eGFR of > 60 mL/min (*n* = 329). The number of patients with CKD after 1 year was 40 (12.16%) and the CSA-CKD score achieved a fair discrimination with an AUC of 0.737 (95% CI 0.657–0.817), which was similar to the original study validation cohort (AUC 0.78, 95% CI 0.72–0.83). Calibration was acceptable with a Chi-square test result of 2.444 and *p* = 0.485 (**Figure 3**).

**Figure 4:**
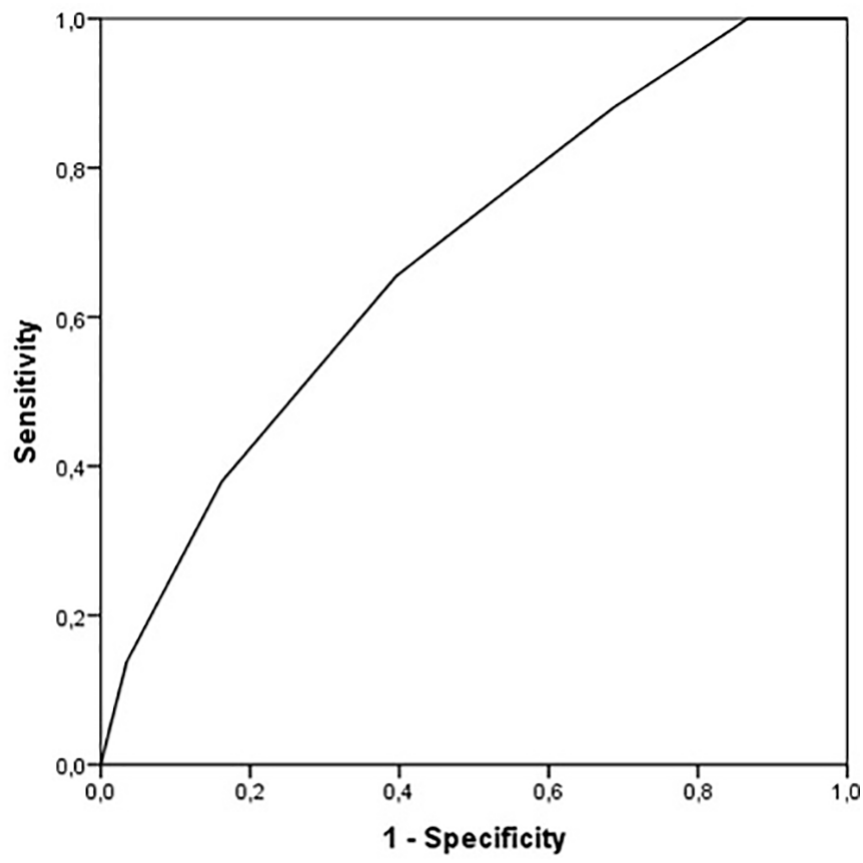
Receiver operating characteristic curve of CSA-CKD score for MAKE. MAKE, major adverse kidney events.

We then assessed the performance of the CSA-CKD score in the overall cohort to assess the likelihood of MAKE after 1 year. The median CSA-CKD score was higher in patients who had experienced MAKE after 1 year [3 (2–4) vs. 2 (1–3), *p *< 0.001). Discrimination fell, with an AUC of 0.682 (0.611–0.754), but calibration was similar (*p* = 0.489) (**Figure 4**).

### 3.4 Risk of 1 year new-AKI episodes

During the 1-year follow-up visit, only 14 patients presented with a registered new AKI episode. Although experience of CSA-AKI was more common in patients who presented with a second AKI during the 1-year follow-up visit (57.1% vs 35.6%), no statistically significant association was found (**Table 6**).

**Table 6:** Univariate analysis of CSA-AKI as a risk factor for new AKI during the 1-year follow-up visit.

	No 1-year AKI (*n* = 390)	1-year AKI (*n* = 14)	OR (95%CI)	*p*-value
Any-stage CSA-AKI	139 (35,6)	8 (57,1)	1,818 (0.499–6.624)	0.365
Long CSA-AKI (> 3 days)	54 (13.8)	4 (28,6)	1.154 (0.249–5.353)	0.855
RRT CSA-AKI	8 (2,1)	2 (14.3)	5.130 (0.79–33.3)	0.087

CSA-AKI, cardiac surgery associated acute kidney injury; RRT, renal replacement therapy; OR, odds ratio.

## 4 Discussion

In this retrospective unicentric study, we evaluated the risk of MAKE after CSA-AKI in a Spanish cohort, and the utility of the CSA-CKD score in the prediction of MAKE after discharge. Any-stage CSA-AKI was the only variable associated with the outcome when analyzed in a multivariate analysis with baseline characteristics of the patients. The CSA-CKD score had acceptable discrimination (AUC 0.737) for the prediction of CKD (eGFR < 60), but the AUC decreased to 0.682 for the prediction of MAKE after 1 year.

GFR generally declines at a rate of 1 mL/min/year (9), but in our cohort we observed median declines of 3 mL/min/year and 8 mL/min/year in patients who did not and did experience CSA-AKI, respectively. Patients who undergo cardiac surgery are at an increased risk of losing kidney function, probably because of their comorbidities (for example, we found that a high percentage of patients who underwent cardiac surgery had diabetes and were hypertensive), but this risk is significantly increased when CSA-AKI occurs (*p *= 0.024). In that regard, Reyden et al. studied a cohort of 29,330 patients who underwent primary isolated CABG in Sweden, with a mean follow-up period of 4.3 years, and found that the risk of end-stage chronic kidney disease (ESCKD) was significantly increased for any-CSA-AKI stage compared with non-CSA-AKI patients, also when stratified by preoperative renal function (10).

Previous studies have focused on the risk of CKD (an eGFR of < 60 mL/min) in this population 1 year after cardiac surgery, but recent evidence shows that defining worsened renal function as a decline of ≤ 25% in eGFR can help to identify patients that can develop CKD in later years, or patients who already have CKD and whose episodes of CSA-AKI could accelerate the decline of their renal function (8). Legouis et al. studied a cohort of 4,791 patients and observed that patients without pre-existing CKD (regardless of their AKI stage) were associated with a risk of *de novo* CKD after fully recovering from an AKI episode after cardiac surgery, and, based on this finding, they developed a CSA-CKD score to identify at-risk patients (5, 7). It is important to note that excluding patients with an eGFR of < 60 mL/min prevents clinicians from identifying patients who can rapidly progress to ESKD and who may benefit from nephrology follow-up. This is particularly important in cardiac surgery as the percentage of patients with pre-existing CKD is increasing, alongside increased rates of patient comorbidity. For instance, in our cohort almost 20% of the patients had pre-existing CKD. Another study, conducted by Ishami et al., included 29,388 individuals who underwent cardiac surgery. They found that a creatinine increase, defined as either none (0%) or as class I (1%-24%), II (25%-49%), III (50%-99%), or IV (100%) was associated, in a graded manner, with an increased risk of incident CKD, CKD stage progression, and mortality (11). This study also gives more weight to the categories of CKD than to the percentage of GFR decline itself. To our knowledge, the present study is the first that focuses on the impact of CSA-AKI on MAKE, with a special focus on the relative reduction of eGFR in line with current AKI research.

Interestingly, the risk of MAKE in our cohort was not associated with age or sex. This is always a major concern when studying eGFR decline, because the CKD-EPI formula includes not only sCR levels but also age and sex (12). Moreover, we did not find differences in the risk of MAKE between the diabetic and non-diabetic populations, which could be explained by the high comorbidity of the whole cohort, which had a median Charlson Comorbidity Index of 4.

Providing information about AKI episodes is key not only to attempts to change the natural history of AKI to CDK transition, but also to the introduction of strategies that identify patients at increased risk to determine which patients may benefit from a nephrology or primary-care follow-up. In that regard, patients in which sCr levels do not return to baseline levels at discharge could be considered candidates for specialist follow-up. However, we must take into account that hyperfiltration after AKI, changes in distribution volume, and loss of muscle mass during long hospital admissions may also decrease creatinine values, and therefore that a large percentage of patients could be lost (13, 14). Interestingly, low sCr levels have been associated with higher mortality rates as a result of malnutrition. On the contrary, when using cystatine C, a biomarker that is independent of muscle metabolism, there is a linear rather than a U–shaped association between eGFR and adverse events (13). The use of cystatin-C may not always be possible, but the measurement of creatinine clearance could be a way to identify patients with persistent kidney dysfunction after CSA-AKI (15). Studies of biomarkers in AKI have mainly been conducted by intensivists and have focused on short-term outcomes. In this field, only a secondary analysis of the Sapphire study for NephroCheck^®^ ([TIMP-2]×[IGFBP7]), known as the cell cycle arrest biomarker, showed that a result of >2 was equivalent to AKI stage progression on the risk of ESKD or death at 9 months (16).

Tools such as the CSA-CKD scoring system developed by Legouis et al. show promise as simple ways to identify patients at risk of kidney disease progression (7). In our study, we first tried to assess if the score had fair discrimination for CKD, as was first described in its original study. We found that the AUC value for CKD in patients without pre-existing CKD was 0.737 (95% CI 0.657–0.817), similar to the validation cohort of the original study (0.78 [95% CI 0.72–0.83]). On the other hand, when analyzing AUC for MAKE in the overall population, the AUC value decreased to 0.682 (95% CI 0.611–0.754). In our study we used the CKD-EPI formula, since it is currently the formula with the most international endorsement. Legouis et al. used the MDRD formula for the estimation of basal GFR in patients without CKD, but it has been proven that this formula has worse precision for eGFRs of 60–90 mL/min, and in that scoring system patients received 1 point for eGFR < 80 ml/min. We believe that multicenter studies are needed to create a new scoring system that focuses on MAKE and uses CKD-EPI as the formula for eGFR estimation (17, 18).

However, after patients at risk of MAKE have been identified, there is still no robust data about the benefits of a specific nephrology follow-up compared to standard care. The first randomized controlled trial investigating this was published in 2021 (19). Patients who survived severe AKI stage 2 or 3 were enrolled and randomized to receive either comprehensive or standard care for 12 months. The comprehensive group comprised a multidisciplinary team that included nephrologists, nurses, nutritionists, and pharmacists. The primary outcome was feasibility and the secondary outcomes included incidence of MAKE, renal function, and albuminuria rate at 12 months. They accomplished the primary feasibility outcomes; for the secondary outcomes they found statistically significant differences only in albuminuria rate. However, blood pressure was better controlled in the comprehensive group.

Our study has some limitations. First, the albuminuria rate was not assessed because data were not available. It is known that post-AKI proteinuria is associated with kidney disease progression and, even in patients without changes in eGFR at 1 year, it is considered a sequela of AKI (20). Second, owing to the nature of this study, almost 7% of the original cohort were lost to follow-up and therefore could not be included in the final analysis. Third, the lack of association between CSA-AKI and new AKI episodes could also be due to the nature of the study, as only 14 new AKI episodes were registered because of the short and retrospective follow-up. Finally, this is a unicentric retrospective study that provides information about the increased risk of MAKE after CSA-AKI, but multicentric and prospective studies are needed to confirm our results and create a scoring system that tries to identify patients at risk of MAKE.

In conclusion, based on our results, any-stage CSA-AKI is associated with MA; however, the development of further scoring systems that help clinicians to identify at-risk patients is needed so that appropriate patient follow-up can be provided.

## Data availability statement

The raw data supporting the conclusions of this article will be made available by the authors, without undue reservation.

## Ethics statement

The Ethics Committee of our institution approved the study (Reg. HCB/2019/0959). Written informed consent from the patient or patient’s legal guardian/next of kin was not required to participate in this study in accordance with the national legislation and the institutional requirements.

## Author contributions

Research idea and study designs: AM and EP. Data acquisition: AM, VE, and AL. Data analysis and interpretation: AM. Supervision and mentorship: EP. IR, PM, CI, MB, ES, LQ, MC, GP, and EQ contributed important intellectual content during manuscript drafting. All authors contributed to the article and approved the submitted version.
