# Supplementary material for: Impact of cardiac surgery associated acute kidney injury on 1-year major adverse kidney events
Source: Front Nephrol. 2023 Apr 24;3:1059668. doi: 10.3389/fneph.2023.1059668 (PMC10479748; doi:10.3389/fneph.2023.1059668)
Supplement: Supplementary file 1 [file Table_1.docx]

SUPPLEMENTARY MATERIAL

Supplementary table 1. Intraoperative variables and scores of AKI/surgical risk

|  | **N (%)/ median (IQR)** |
| --- | --- |
| **Blood transfussion** | 102 (25.2) |
| **Vasodilator agents use** | 140 (34.7) |
| **Dobutamine use** | 173 (42.8) |
| **Furosemide** | 99 (24.5) |
| **Vasoconstrictor agents use** | 234 (57.9) |
| **CPB time (min)** | 90 (70-113) |
| **Ischemia time (min)** | 63 (50-83.25) |
| **Euroscore II** | 1.73 (1.08-2.96) |
| **CCS** | 0.4 (0.4-1.8) |
| **Leicester Score** | 18.3 (10,94-30.63) |

IQR: interquartile range; CPB: cardiopulmonary bypass; CCS: Cleveland Clinic score

Supplementary table 2. CSA-AKI characteristics

| **AKI Stages** | **Days between surgery and AKI start** |
| --- | --- |
| AKI stage 1: 93 (63,3%)🡪43 met only the “>0.3 mg/dL in 48h” criteria (46.2%)  AKI stage 2: 33 (22.4%)  AKI stage 3: 21 (14.3%)🡪10 with dialysis requirement (47,6%) | Median time from surgery to AKI: 1 (1-2)  First 24h: 92 (62,6%)  48h: 35 (23.8%)  72h: 10 (6.8%)  >72h: 10(6.8%) |
| **AKI duration** | **Dialysis technique** |
| Median duration time (days): 3 (1-6)  24h: 46 patients (31.3%)  48h: 22 patients (14.9%)  72h: 19 patients (12.9%)  >72h: 60 patients(40.8%) | *Intermitent hemodialysis: 6 patients  CRRT: 2 patients  Both: 2 patients  *Median hemodialysis sessions: 2 (IQR 1-4)  Median CRRT treatment (days): 4,5 (1,75-11,75) |

*CSA-AKI: cardio surgery associated acute kidney injury; AKI: acute kidney injury; CRRT: continuous renal replacement therapy*
